# No-Needle, Single-Visit Adult Male Circumcision with Unicirc: A Multi-Centre Field Trial

**DOI:** 10.1371/journal.pone.0121686

**Published:** 2015-03-30

**Authors:** Peter S. Millard, Norman D. Goldstuck

**Affiliations:** 1 University of New England, Portland, Maine, United States of America; 2 Faculty of Medicine and Health Sciences, Stellenbosch University and Department of Obstetrics and Gynaecology, Tygerberg Hospital, Cape Town, South Africa; Cardiff University, UNITED KINGDOM

## Abstract

**Background:**

Voluntary medical male circumcision (VMMC) is a priority HIV preventive intervention. Current adult circumcision methods need improvement.

**Methods:**

Field trial in 3 primary care centres. Minimally invasive VMMC using the Unicirc instrument following topical lidocaine/prilocaine anesthetic. Men were followed up at 1 and 4 weeks.

**Results:**

We circumcised 110 healthy volunteers. Two men complained of transient burning pain during circumcision, but none required injectable anaesthesia. Median blood loss was 1ml and median procedure time was 9.0 min. There were 7 (6.3%) moderate complications (5 (4.5%) post-operative bleeds requiring suture and 2 (1.8%) post-operative infections) affecting 7 men. No men experienced significant wound dehiscence. 90.4% of men were fully healed at 4 weeks of follow-up and all were highly satisfied.

**Conclusions:**

Use of topical anaesthesia obviates the need for injectable anesthetic and makes the Unicirc procedure nearly painless. Unicirc is rapid, easy to learn, heals by primary intention with excellent cosmetic results, obviates the need for a return visit for device removal, and is potentially cheaper and safer than other methods. Use of this method will greatly facilitate scale-up of mass circumcision programs.

**Trial Registration:**

ClinicalTrials.gov NCT02091726

## Introduction

Voluntary medical male circumcision (VMMC) reduces female-to-male transmission of HIV by 38% to 66% over 24 months.[1] Almost 6 million men have been circumcised in 14 priority African countries of Eastern and Southern Africa, with a goal of 20 million by 2016.[2] If this goal is met, more than 3.4 million new HIV infections can be prevented in Southern and Eastern Africa with an estimated net savings of US$16.5 billion between 2011 and 2025.[3]

We have shown in a previous randomized controlled trial that VMMC, under local injectable anaesthetic, using the Unicirc instrument to excise the foreskin and sealing the wound with cyanoacrylate tissue adhesive is faster, easier, and has superior cosmetic results compared to open surgical circumcision.[4–5] A subsequent case series of 50 Unicirc circumcisions confirmed the rapidity, safety, and excellent cosmetic results of Unicirc circumcision under local anaesthesia.[6]

This study reports on a field trial of needle-free VMMC using topical lidocaine/prilocaine anesthetic, excising the foreskin with the Unicirc instrument, and sealing the wound with tissue adhesive.

## Methods

The protocol for this trial and supporting TREND checklist are available as supporting information; see S1 TREND Checklist and S1 Protocol.

### Trial design

This was a multi-centre field trial with two sites in Cape Town and one site in Marikana, South Africa. Men were recruited via posters and word of mouth in the respective primary care clinics. We obtained written informed consent from each participant. The South African Medical Association's Research Ethics Committee (SAMAREC) approved the study and the informed consent. The ClinicalTrials.gov identifier is NCT02091726. The study took place between January 20 and July 4, 2014. There were no deviations from the study protocol.

### Participants

WHO, in the Framework for Clinical Evaluation of Devices for Adult Male Circumcision (2009)^[^ ^7^ ^]^ states: “Studies involving about 100 men (range 50 to 300) are suggested as a compromise between assessing safety, documenting the presumed advantages of the new method, and ensuring rapid progress through the different stages of clinical assessment. The exact choice of endpoint will be determined by the expected advantages of the new device over conventional surgery, but the total operation time is likely one key measure by which to compare the approaches.”

We therefore aimed for a sample size of approximately 100.

Healthy uncircumcised men at least 18 years of age were eligible for the study. Exclusion criteria were concurrent illness, history of bleeding disorder, past reaction to local anesthetic, infection, or penile abnormality which could complicate circumcision.

Participants received HIV prevention counseling. We offered HIV testing, but we did not request testing as a study prerequisite, per SAMAREC guidelines. Participants were advised to abstain from sexual intercourse until the wound was completely healed and for at least 4 weeks after the circumcision. Condoms were made freely available.

### Intervention

Three generalist physicians (GPs) performed the circumcisions in individual consultation rooms in their own primary health care clinic. Two of the GPs (SN, ZP) had previous experience with the Unicirc; SKN trained on the instrument for this case series.

#### Unicirc with cyanoacrylate skin adhesive

**Fig 1:**
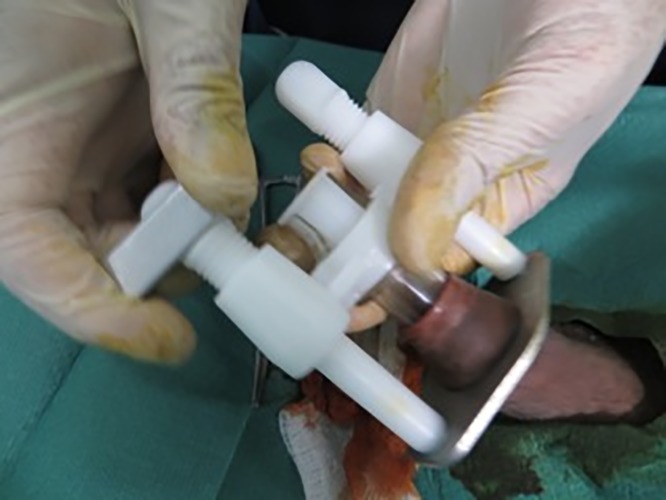
Unicirc placement.

**Fig 2:**
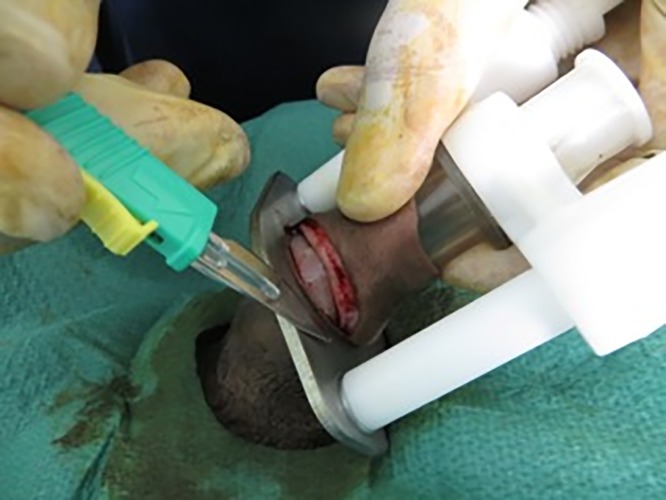
Excising the foreskin.

**Fig 3:**
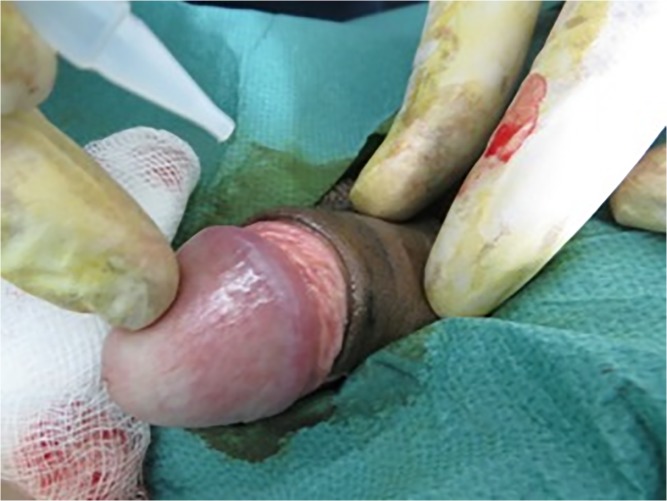
Applying cyanoacrylate adhesive.

The Unicirc is a plastic and metal single-use-only disposable instrument designed in South Africa. For this study, we used the same instrument that we used in our prior case series,[6] which was a slight modification from the instrument used in our randomized controlled trial.[4] Prior to use, the instruments were gas sterilized in sealed packages. After applying a topical mixture of lidocaine/prilocaine (EMLA cream) to the glans and foreskin, we waited approximately 30 minutes before applying the Unicirc instrument (Fig. 1). We left the instrument in place for 5 minutes before excising the foreskin with a surgical scalpel (Fig. 2). After removing the instrument, we sealed the apposed skin-mucosal edges with cyanoacrylate skin adhesive (Fig. 3). We used four different Unicirc sizes (diameters) in this study: 2.6 cm, 2.9 cm, 3.2 cm, and 3.5 cm.

We covered the wound with an adherent tape (Hypafix) and absorbent gauze. We asked the volunteer to remove the absorbent gauze at day two, to keep the wound dry, and to leave the adherent tape in place for two weeks.

All men were observed for 20 minutes after the procedure. Subjects were given written post-operative instructions and cellular telephone contact information of the doctor.

### Outcomes

Outcome definitions are shown in Table 1.

**Table 1:** Outcome definitions.

Endpoint	Definition
Operating time	Time from first clamp on foreskin until dressing placed
Pain assessment using Visual Analog Scale (0–10)	Self-reported pain during first 24 and 48 hours
Blood loss	Quantity estimated by senior surgeon (ml)
Adverse Event	Mild adverse events required no active intervention other than wound pressure for bleeding; moderate events required medical intervention (sutures, antibiotics); severe events required transfusion, hospitalization, or resulted in permanent disfigurement
Wound infection	Empirical diagnosis based on wound swelling, redness, and pain. No bacterial cultures were available
Wound disruption	Length of wound disruption or granulation tissue (< 2cm vs. > 2cm)
Wound fully healed	Completely epithelialized; no superficial ulcerations or granulation tissue present
Cosmetic appearance	Regular: scar line straight without any irregularity
	Irregular: Some irregularity to scar line
	Scalloped: wavy appearane to scar line
Participant satisfaction (5 point Likert scale)	Are you satisfied with your circumcision result? If not, why not? Would you recommend circumcision to friends or relatives?

#### Primary Outcome Measure

Intraoperative time

#### Secondary Outcome Measures

Intra-operative pain; complications (operative and post-operative); time to healing; patient satisfaction; cosmetic result.

#### Adverse events

Key adverse events considered were anaesthetic complications, bleeding, haematoma, infection, wound disruption, problems with urination, subsequent procedures conducted to correct complications, and occupational exposure to blood and body fluids. We used standardized definitions to grade adverse events as mild, moderate, or severe, using the WHO Framework.[7] In brief, adverse events were categorized as mild if they required little or no intervention (e.g. mild wound disruption or slight bleeding), moderate if they required active treatment (e.g. antibiotics or suturing), or severe if they required transfusion or hospitalization, or resulted in permanent damage.

### Follow-up

Follow-up was at seven days and four weeks. For those men who were not completely healed by four weeks, we conducted an additional six-week follow-up visit.

### Outcome assessment

Complications and wound healing outcomes were assessed by the GPs.

## Results

### Participant flow

**Fig 4:**
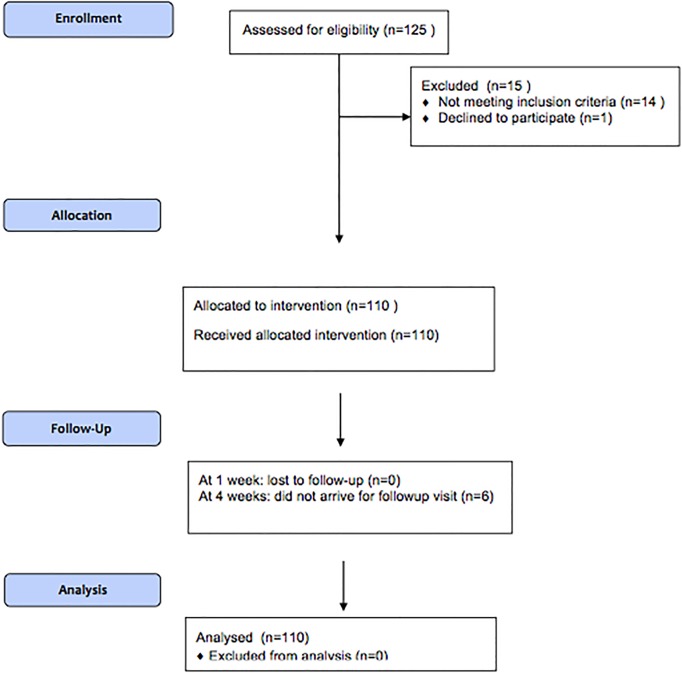
Flow diagram.

A total of 125 men volunteered to participate and 110 men underwent circumcision (Fig. 4). One volunteer completed the informed consent but did not arrive for his circumcision. We excluded two men with hypospadias, one man with a scarred frenulum, and 12 men with phimosis. All participants were circumcised using topical anaesthetic and Unicirc. Per WHO guidelines, the doctors decided that 2 men with tight (but not scarred) frenulums could participate. Each of the 2 frenulectomies was done and sutured under topical anaesthetic as a separate procedure immediately prior to the circumcision. The sutures remained in place, and did not affect healing of the subsequent Unicirc circumcision, which was done without sutures and did not result in bleeding.

### Baseline data

The baseline characteristics of the participants are shown in Table 2. The median age was 29.5 (IQR 23.5, 37.5) years. The large majority of men gave improved hygiene as their motivation for circumcision and few were motivated by HIV prevention.

**Table 2:** Baseline characteristics.

	Unicirc/adhesive
	N = 110
**Age (yrs)**, n (%)	
*18–25*	38 (34.5)
*26–35*	41 (37.2)
*36+*	31 (28.2)
**Religion**, n (%)	
*Christian*	101 (92.0)
*Muslim*	7 (6.4)
*No religion*	2 (1.8)
**Employment**, n (%)	
*Employed*	41 (37.3)
*Self-employed*	3 (2.7)
*Student*	1 (0.9)
*Unemployed*	65 (59.1)
**Reason for circumcision**, n (%)	
*Hygiene*	86 (78.2)
*Reduce HIV risk*	9 (8.2)
*Social/religious*	10 (9.1)
*Appearance*	5 (4.6)

### Outcomes analyzed

Operative outcomes are shown in Table 3. Two of the participants felt burning when the instrument was tightened but none required injectable anaesthesia. One participant had a partial phimosis and required a dorsal slit before applying the Unicirc. No participant required intraoperative suturing and there were no intraoperative complications. The median operative time time was 9 minutes (range: 7–15 min) and blood loss was minimal (range: 0–3 ml).

**Table 3:** Intraoperative outcomes.

	Unicirc
	N = 110
**Center**	
*Cape Town site 1 (ZP)*, *n (%)*	12 (11)
*Cape Town site 2 (SN)*, *n (%)*	80 (73)
*Marikana (SKN)*, *n (%)*	18 (16)
**Intraoperative suturing**, *n* (%)	0
**Frenulectomy performed**, *n* (%)	2 (1.8)
**Intraoperative time (min)**, median (IQR)	9 (9, 10)
**Estimated blood loss (ml)**, median (IQR)	1 (1, 1)
**Pain on crushing foreskin**, n (%)	2 (1.8)

IQR = Interquartile range

Adverse events are shown in Table 4. There were no serious complications. The overall rate of moderate complications was 6.3%, (5 (4.5%) post-operative bleeds requiring suture and 2 (1.8%) post-operative infections) affecting 7 men. There was no association between the surgeon’s experience and complication rate. All bleeding episodes occurred after the dressing was placed and were treated before the volunteers left the site with haemostatic suture of the bleeding site and closure of the part of the wound that was disrupted by the haemostasis. After suturing, the wound was covered by a dry dressing. There was no clinically significant wound dehiscence > 2 cm.

**Table 4:** Adverse Events.

	Unicirc
	N = 110
**Serious post-operative complication**, n	0
**Post-operative bleeding**, n (%)	
*Mild (dressing only)*	1 (0.9%)
*Moderate (sutured)*	5 (4.5%)
**Haematoma**, n	0
**Post-operative infection** *(antibiotic required)*, n (%)	2 (1.8%)
**Wound disruption at 1 wk**, n (%)	
*< 2 cm length*	5 (4.5%)
*> 2 cm length*	0

**Fig 5:**
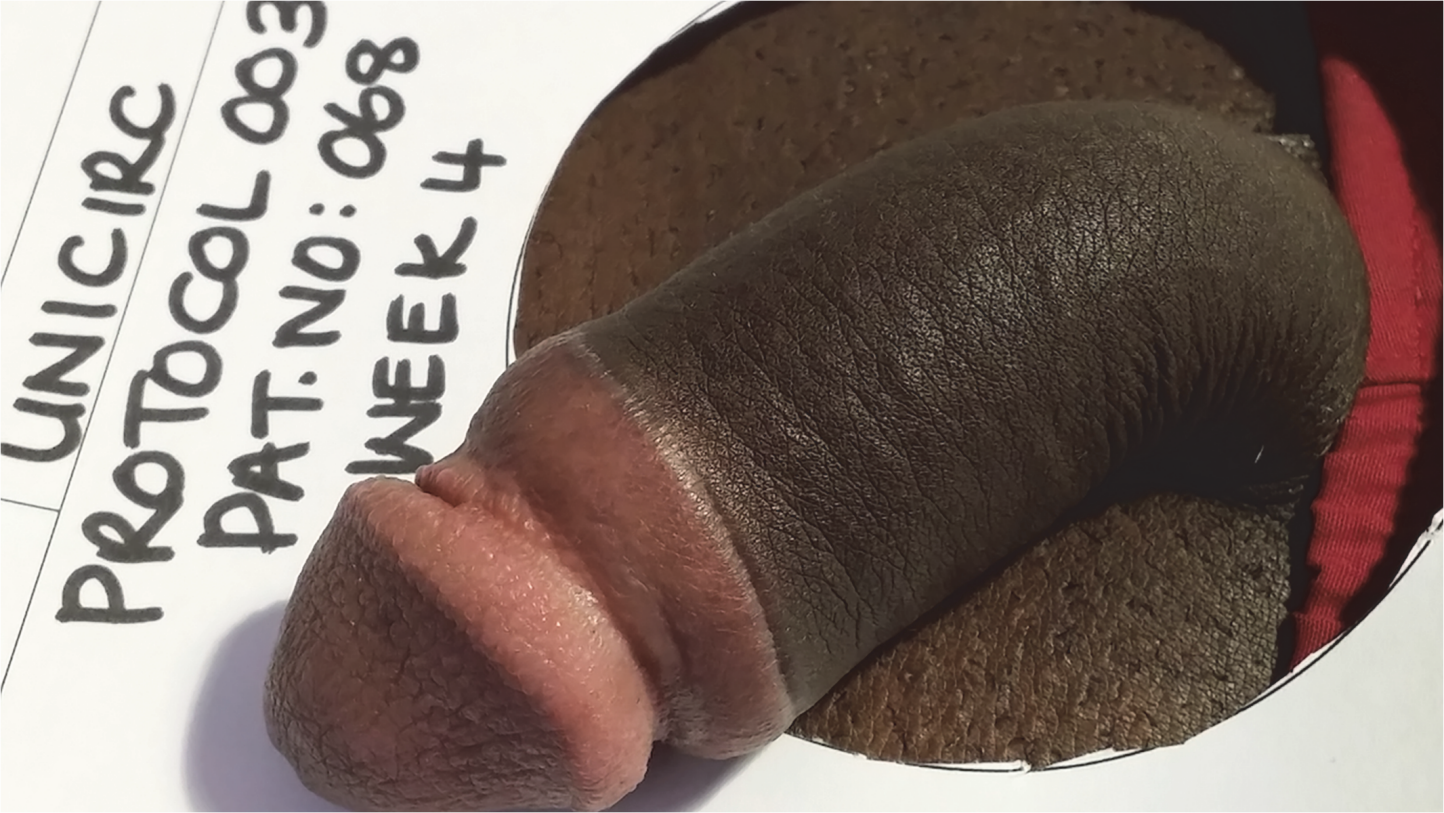
Four-week follow-up.

Wound healing at 4 weeks, participant satisfaction, and cosmetic results are shown in Table 5. All wounds were completely healed by 6 weeks. The cosmetic result was excellent (Fig. 5) in all but one patient who had required suturing for post-operative bleeding.

**Table 5:** Outcomes at 4 weeks.

	Unicirc
	N = 104
**Wound fully healed at 4 weeks**, n (%)	94 (90.4%)
**Satisfaction** ^**a**^, n (%)	
*Very satisfied*	103 (100%)
*Not satisfied*	0
**Recommendation** ^**a**^, n (%)	
*Recommend highly*	103 (100%)
*Not recommend*	0
**Cosmetic results** ^**b**^, n (%)	
*Regular*	101 (99%)
*Irregular*	0
*Scalloped*	1 (1%)

^a^In one case, the interviewer did not ask either the question about satisfaction or recommendation

^b^In 2 cases, the doctor failed to assess the cosmetic result

## Discussion

VMMC is an essential intervention to prevent female-to-male HIV transmission. Interesting, in a country with a great deal of publicity about reducing HIV infection through VMMC, only 8.2% of the volunteers in this study indicated that partial HIV protection was their main reason for desiring circumcision. The challenge for areas with high HIV prevalence is to provide safe and cost-effective VMMC. In order to more effectively scale-up services, we require surgical techniques that are rapid, easy to learn, can be performed with standard instruments, are inexpensive, result in few complications, and provide excellent patient satisfaction and cosmetic results.

The Unicirc method is safe, rapid, easy to perform, requires only one visit for completion, and has excellent cosmetic results. We have now shown another considerable advantage: it requires no injectable local anesthetic. Because it requires neither an injection nor suturing, Unicirc is potentially safer than other methods.[4]

Our first study,[4] was marred by the need for intra-operative suturing in 17% of participants. We attributed this to a defect in the manufacturing process and the device developer subsequently corrected this problem. No participants required intraoperative suturing in our second study,[6] nor in this, our third Unicirc study.

Healing is delayed using plastic ring devices such as Prepex and Shang Ring, because they heal by secondary intention. As a consequence, a major drawback of plastic rings may be an increased probability of HIV transmission during the healing period. Like the Gomco/adhesive method,[8] Unicirc/adhesive heals by primary intention. This is the fourth study using one of these two methods (N = 347 assessed for healing at 4 weeks) showing healing times virtually identical to open surgical circumcision with suturing. This is to be expected, since the average time to healing by primary intention is constant.

Compared to the approximately 20 minutes required for a surgical circumcision, the 9-minute operating time includes 1–2 minutes to place the instrument, 5 minutes of waiting after the Unicirc is applied for the crushing action to take place, and 2–3 minutes to excise the foreskin, remove the instrument, and apply the adhesive. Using WHO’s MOVE model of task-sharing,[9] the 5 minute waiting time could be utilized for placing (or removing) the instrument on other patients. The actual time-savings using the Unicirc/adhesive technique are therefore likely to be greater than reflected by these 9 minutes, and should substantially reduce overall cost and assist in mass scale-up.

Unlike plastic ring methods, the Unicirc method doesn’t require a second visit for device removal, saving time and staff resources that can be used to circumcise additional men.

The cost of expendable materials is similar to other methods. The cost of tissue adhesive approximates the cost of suture, and Unicirc requires fewer disposable instruments (only one haemostat and one scalpel) than other techniques. The market price has not been set for Unicirc, but would probably be similar to that of plastic ring devices. Because no follow-up visits are required, we expect there to be significant cost savings compared to plastic ring devices.

No one technique will be suitable for all settings. The Unicirc method is ideal for outpatient settings where large numbers of circumcisions are performed by mid-level staff using the MOVE model, or for use by private practitioners who have basic surgical skills and wish to add circumcision services to their practice. It is not suitable for rural clinics without the ability to suture.

This field study was performed by moderately experienced GPs in their own practices. It shares the same advantages and disadvantages of other field studies: it is more indicative of real-world conditions, but outcomes are not as tightly controlled as in a clinical trial. For example, there was no independent, objective measure of wound healing outcomes, which were assessed by the surgeons themselves. Given the high follow-up rates and the easy available of the practitioners via cell phone, we think it unlikely that we missed any adverse events.

## Conclusions

This study has important implications for the scale-up of VMMC services. Using topical anaesthesia, excising the foreskin after applying the the Unicirc instrument for 5 minutes and sealing the wound with cyanoacrylate tissue adhesive in adults is rapid, easy to learn, heals rapidly by primary intention with excellent cosmetic results, and is potentially cheaper and safer than other methods. Use of this new method will greatly facilitate scale-up of mass circumcision programs.

## Supporting Information

S1 Protocol Trial Protocol. (PDF)

S1 TREND Checklist TREND Checklist. (PDF)
