# Supplementary material for: No-Needle, Single-Visit Adult Male Circumcision with Unicirc: A Multi-Centre Field Trial
Source: PLoS One. 2015 Mar 30;10(3):e0121686. doi: 10.1371/journal.pone.0121686 (PMC4379010; doi:10.1371/journal.pone.0121686)
Supplement: S1 TREND Checklist — (PDF) [file pone.0121686.s002.pdf]

UNICIRC Protocol Phase 4 research data collection: Application for a registry.

Voluntary Adult Male Circumcision

e-mail: management@simunyehealthcare.com
www.simunyehealthcare.com
telephone: 021 3912662
facsimile: 021 3912663

Date: 14 December 2013

Dear Colleagues,

Please accept the attached documents for review by the South African Medical Association Research and Ethics committee.

In brief, this is an application for a Phase 4 research registry to collect data from further field trials of this proven technique. The UNICIRC Protocol 001 and 002 were completed in June 2013 and October 2013, which complication rates the same (001) or less than (002) the surgical method.

The results of our our 2 Unicirc studies are scheduled for publication in the SAMJ in January 2014. The results are also listed on clinicaltrials.gov, the site of the US National Health Institute.

Population: Healthy men of at least 18 years of age who desire male circumcision
Intervention: Unicirc disposable, single-use-only instrument plus tissue adhesive
Follow-up visits: 7 days and 28 days. Optional 2 and 14 day follow-ups, depending on site. Optional 42 day follow-up if not completely healed by 4 weeks.
Primary outcome: time required for procedure
Secondary outcomes: complications (operative and post-operative); time to healing; cosmetic result.

Although medical circumcision is a very safe procedure, minor complications (principally post-operative bleeding and infection) occur in up to 10% of cases. Serious complications, which require hospitalisation or result in permanent disability, are extremely rare with medical circumcision.

The Doctors performing the circumcisions will be fully qualified and skilled in circumcison. They will play no role in the management or analysis of data, the reporting of the results, or the decision to publish the results.

Sincerely,

Cyril Parker
MBChB(Wits), FRACGP(Australia), DA(SA), Cert. Dermatology (Australia)
HPCSA / MP No 0248495
MPS No SA/10601

UNICIRC Protocol Phase 4 research data collection: Application for a registry.

Voluntary Adult Male Circumcision

SUMMARY

Voluntary medical male circumcision (VMMC) is a priority preventive intervention and South Africa is currently scaling up VMMC.

Open surgery is the most common circumcision technique used in Africa, but it has a number of drawbacks. According to the Framework for Clinical Evaluation of Devices for Adult Male Circumcision (WHO, 2011): *"WHO and other health authorities wish to identify one or more devices that (a) would make the VMMC safer, easier, and quicker; (b) would have more rapid healing than current methods and/or might entail less risk of HIV transmission in the post-operative period; (c) could be performed safely by health-care providers with a minimal level of training; and (d) would be cost-effective compared to standard surgical methods for male circumcision scale up."*

Because of difficulties and small risks entailed in using sterilizable instruments, WHO prefers disposable, single-use-only instruments be used for circumcision. **We have recently shown that VMMC using the Unicirc disposable single-use-only instrument coupled with tissue adhesive meets WHO criteria for the ideal method: it is faster, easier to learn, safer for both surgeons and patients, heals sooner, has excellent cosmetic results, and is more cost effective than any other currently available technique. (Millard SAMJ 2014).**

A Phase 4 registry for field trials will give further information on this proven method and enable the South African health system to more effectively scale-up circumcision services. We propose data collection of Unicirc Circumcisions with tissue adhesive at several field sites in accordance with WHO recommendations concerning sample size found in the Framework for Clinical Evaluation of Devices for Adult Male Circumcision.

The Unicirc instrument has the identical mechanism of action as the Gomco instrument, an FDA-approved surgical instrument widely used in the US since 1935. The UNICIRC instrument qualifies for FDA approval through the 510(K) mechanism, showing substantial equivalence to the Gomco in its function. The functionality and method of circumcision, with circumferential crushing, has been studied extensively in infants, children and adults for many years.

Tissue adhesive is widely used in multiple areas of medicine; specifically, multiple RCTs have shown cyanoacrylate tissue adhesives to be superior to suture closure in VMMC and we have re-confirmed this in our two recent studies.

Population: Healthy men who desire male circumcision
Study design: Phase 4 field trials
Intervention: Unicirc circumcision instrument plus tissue adhesive
Follow-up visits: 7 days and 28 days. Optional follow-ups at 2 days and 14 days, depending on site. 42 day follow-up if not completely healed by 4 weeks.
Primary outcomes: Intraoperative time required for procedure
Secondary outcomes: Complications (operative and post-operative); time to healing; patient satisfaction; cosmetic result.

UNICIRC Protocol Phase 4 research data collection: Application for a registry.

Voluntary Adult Male Circumcision

The study is not designed to study HIV transmission, and therefore HIV status is not relevant to the study. For this reason, we will not ask participants questions concerning their HIV status, nor will we require HIV testing as part of the study protocol. However, the participants will be offered HIV Counseling and Testing.

1.0 HYPOTHESES AND STUDY OBJECTIVES

OBJECTIVE: To further quantify the effectiveness of a minimally-invasive surgical circumcision technique for men, which is easy to learn and perform, is safe, and is associated with high patient satisfaction and excellent cosmetic results.

SPECIFIC OBJECTIVES: To quantify the time taken to perform a circumcision with the Unicirc device, ease-of-learning, ease-of-use, time to healing, and patient satisfaction.

The study is not designed to study HIV transmission, and therefore HIV status is not relevant to the study. For this reason, we will not ask participants questions concerning their HIV status, nor will we require HIV testing as part of the study protocol. However, the participants will be offered HIV Counseling and Testing.

2.0 BACKGROUND:

Our prior three studies showed that excising the foreskin after applying either the Gomco or Unicirc instrument and sealing the wound with tissue adhesive has important advantages over open surgical circumcision.(1, 2) It requires much less operative time, is easier to perform, has better cosmetic results, and is potentially safer because it does not require suturing. However, the Gomco instrument has drawbacks that make it less than ideal for mass circumcisions in resource-limited settings. Given the history of transmission of blood-borne infections from poorly sterilized instruments, WHO prefers disposable devices, but the Gomco instrument is a re-usable, sterilizable instrument. Given the fact that the Gomco instrument consists of three different parts, mismatching of parts from different-sized instruments or different manufacturers may potentially cause complications.

The Unicirc shares the advantages of the Gomco instrument, but it overcomes the above drawbacks. It is a metal and plastic instrument which is designed with threads that self-destruct after a single use, definitively preventing re-use. Because it is pre-packaged, mismatching of parts is not an issue.

Our previous studies were conducted using generalist doctors who were moderately experienced in open surgical circumcision but had not previously used the Gomco or Unicirc instruments. Operative times were much less with with Unicirc vs surgical VMMC (13 vs. 22.6 min). Using WHO's MOVE model of task-sharing,(3) the actual time savings should be much greater than is reflected by this difference. It takes 1-2 minutes to place the Unicirc instrument, and 2-3 minutes to excise the foreskin, remove the instrument, and apply the adhesive. The 5 minutes of waiting time while the compressive action takes place could well be used in other tasks. The time savings using the Unicirc/adhesive technique are likely to substantially reduce overall cost and assist in mass scale-up.

There were no serious adverse events in any of the prior studies, and post-operative complications were similar in the two groups. In the first Unicirc study, 17% subjects required intraoperative suturing, and there was a non-significant trend toward increased post-operative bleeding and hematoma. We attributed the bleeding to shrinkage that occurred after the injection-moulding of the plastic component of the Unicirc production instruments used in the first study, and this was corrected by minor revisions to the device in order to increase the compressive forces. In the second case series of 50 subjects (reported on

Clinicaltrials.gov), there was no intra-operative suturing required (Table 1).

Because the Unicirc procedure requires no follow-up visits, we expect there to be significant cost savings compared to plastic ring devices, such as the Shang Ring and PrePex. Plastic ring devices also require both an application pack and a removal pack, which contains scissors and a device for removing the ring.

Because the Unicirc/adhesive technique requires no sutures, there is no possibility of a needle-stick injury from a suture needle. All doctors preferred the Unicirc method to the surgical method, citing ease of performance and shorter intraoperative time as advantages of the Unicirc.

2.1 Behavioral disinhibition

This study does not aim to assess the effectiveness of male circumcision to prevent HIV. However, there is some public concern that sexual disinhibition might result from a false sense of security following circumcision that could offset potential protective effects of the procedure. There is no clear evidence that risky behavior is increased following circumcision (4), but educational efforts regarding safe sexual practices will be imparted to all participants of this study. The benefit of lower rates of HIV transmission cannot be guaranteed, and this is not an aim of this research.

2.2. Benefits of circumcision

The benefits of having voluntary male circumcision include:

- Reduced risk of HIV infection and some other sexually transmitted diseases. Male circumcision does not provide complete protection against HIV or other sexually transmitted diseases.
- Reduced risk of cancer of the penis; and, increased cleanliness.

2.3 Techniques for circumcision

There are many surgical techniques for performing circumcision, but few studies have compared techniques for ease of performance, safety, and patient satisfaction. The World Health Organization, in its Manual for Male Circumcision under Local Anesthesia, describes three open surgical techniques (forceps assisted, dorsal slit and sleeve technique), all three of which involve (a) exposure of subcutaneous tissues and (b) suturing for hemostasis and for skin closure. The minimally-invasive technique that will be offered to participants, avoids both exposure of subcutaneous tissues and the need for sutures (5).

Open surgical techniques, which are commonly used for circumcision, require good surgical skills and minor complications are common. In Bailey's prospective study of medical and traditional circumcisions in Kenya, complication rates were 17.7% in the medical circumcision group, and wounds had not healed by postoperative day 60 in 19% of men (6). Bleeding was the most common complication, but other significant complications included infection, excessive pain, pain upon urination, incomplete circumcision requiring additional surgery, and lacerations of the glans and scrotum (6). Studies of open surgical circumcision techniques have also revealed shortcomings in practitioner knowledge and training (10). Subsequent clinical data from better-trained personnel who were trained during and since the conduct of the randomized clinical trials have shown lower complication rates, on the order of 3 to 9%, with complication rates related to the experience and other characteristics of the health provider (Herman-Roloff, AIDS 2010 (Vienna), Poster # MOPE0366). The use of minimally-invasive techniques should further reduce these complication rates.

UNICIRC Protocol Phase 4 research data collection: Application for a registry.

Voluntary Adult Male Circumcision

Before scaling up male circumcision services in Africa, we require fundamental improvements in current circumcision techniques and a careful strategy to minimize unnecessary complications (7). The challenge to provide safe and cost-effective circumcisions in resource-limited settings is great. The key is to have one or more surgical techniques that are easy for generalist physicians and technicians to learn, can be performed with standard instruments, are minimally invasive and cost-effective, result in few complications, and provide excellent patient satisfaction.

2.4 Rationale for data collection.

We have identified a minimally-invasive technique for circumcision which is easy to learn, inexpensive, and which we expect to have low complication rates. The Unicirc disposable instrument is identical in functioning to the (US) FDA-approved Gomco instrument, which has been widely used in the US since 1935.
There is currently enthusiasm about several plastic ring devices, which are placed on the penis and left for one week before they are removed. The Unicirc is *not* one of those devices. Similar to the Gomco instrument, it is a surgical instrument which is left on for only 5 minutes intraoperatively.

Until recently, the main disadvantage of the Gomco instrument circumcision outside of the newborn period has been the need for sutures to reinforce the apposed excision line; the need for sutures has now been obviated by the demonstration that cyanoacrylate tissue adhesives are safe, effective, and superior to sutures in circumcision.

2.4.1 Gomco instrument.

According to WHO (2011), the Gomco instrument has "…an impeccable safety record. In the USA, where it is estimated that well over 1 million neonates are circumcised each year, the Gomco instrument has become the leading instrument used to perform non-ritual male circumcision." There are very few complications from the Gomco method, but mis-matching of parts from different-sized Gomco instruments may cause tearing of tissue (8).

2.4.2 Unicirc disposable instrument

The Unicirc hard plastic disposable instrument has been designed in South Africa for rapid, safe circumcision. The instrument encircles the penis and crushes the foreskin along a circumferential line of 2.5mm. The instrument is left on for 5 minutes to compress the tissues. Identical in function to the Gomco clamp, it temporarily fuses the skin (outer layer) and mucosa (inner layer) together. The foreskin is then cut with a scalpel distal to the compression line, and the instrument is removed. The instrument is designed so that it cannot be reused. Tissue adhesive is then used to seal the apposed skin edges.

2.4.3 Tissue adhesive. A systematic review of randomized controlled trials of tissue adhesive in circumcision showed that it reduces operative time, improves cosmetic result, and increases patient satisfaction when compared to sutures (9). One pediatric study combined the Gomco instrument circumcision with tissue adhesive (10), but other studies used freehand circumcision with CO2 laser or diathermy, which may not be available in resource-limited settings. Adverse effects reported from tissue adhesive are minor and include wound separation, wound inflammation, and inadvertent incorporation of surrounding structures such as scrotal skin and hair into the wound (9).

The following picture shows the Unicirc method of circumcision

UNICIRC Protocol Phase 4 research data collection:
Application for a registry.

Voluntary Adult Male Circumcision

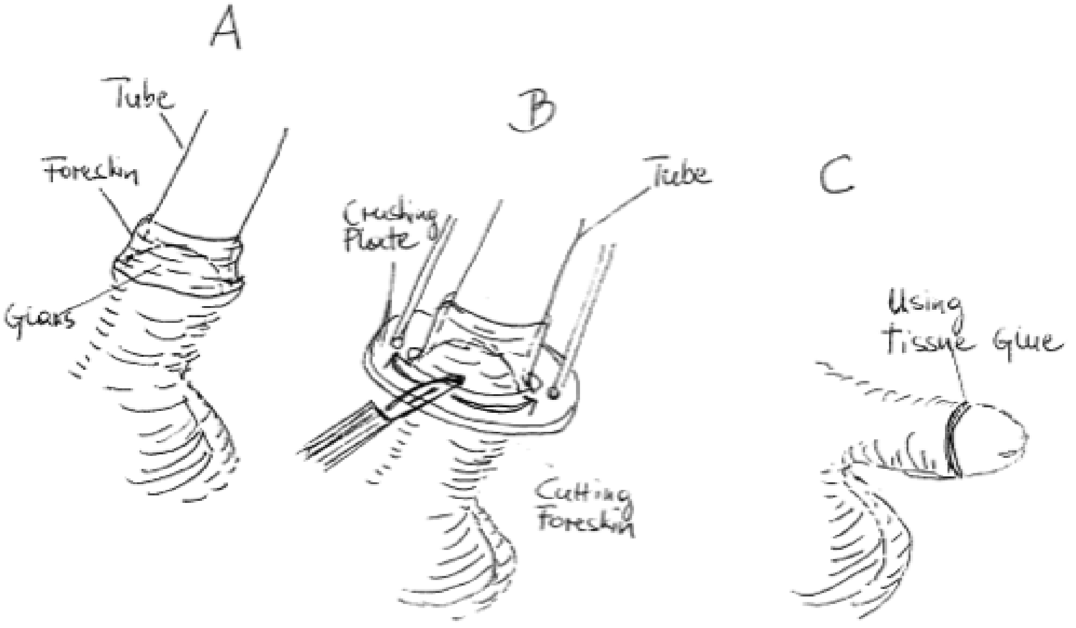

Coupling removal of the foreskin with the Unicirc instrument followed by wound sealing with tissue adhesive will result in a procedure that can be performed by generalist doctors using the same technique in all age groups. Like the Gomco procedure, the incision should bleed rarely, should result in little post-operative swelling, and the adhesive has been shown to be sufficiently strong that the excision line doesn't separate during erections.(11)

3.0 PROJECT STAFF
Normal Clinicians
3.1 Training of project staff
All doctors are fully qualified and skilled in performing male circumcision. Ongoing training in use of the Unicirc disposable instrument will be provided by the Dr. C Parker, who is a qualified physician with 20 years of circumcision experience. Standard Operating Procedures will be in place to ensure consistency between doctors in circumcision technique.

3.2 Changes in protocol
Any changes to project protocol that occur during the project will be accompanied with training.

4.0 RECRUITMENT / CONSENTING
4.1 Participating Project site
The questionnaire and consents will occur in rooms where confidentiality can be assured. The circumcision procedures will occur in a medically equipped minor surgery in rooms that have modern surgical equipment and resuscitation equipment. Only disposable instruments will be used.

4.2 Inclusion / Exclusion Criteria
Inclusion Criteria:
- Healthy men requesting circumcision

- No penile anatomical abnormalities or infections
- Able to provide informed consent to participate
- Willing to participate in all follow-up visits

Exclusion Criteria:
Exclusion criteria will include:
- Current illness
- Penile abnormality or infection which contraindicates or would complicate circumcision
- History of bleeding disorder
- Past reaction to local anesthetic

4.3 Recruitment
Recruitment will be conducted through posters, by advertising in Doctor's rooms, and by word of mouth.

4.4 Performing Circumcisions
The doctors involved will be fully licensed and qualified to perform adult male circumcision; all currently perform VMMC in their practice and all will be taught by Dr. Parker or another highly experienced doctor to apply Unicirc instrument. Only Unicirc circumcision data will be collected.

4.5 Informed Consent
Informed Consent will be discussed in depth with potential participants, and they will be encouraged to ask questions and to discuss the data collection in detail. If participants cannot read the form, it will be read to them in one of 2 local languages (Afrikaans, Xhosa) by an assistant fluent in English and the local language. Participants will sign and date the relevant Informed Consent form(s). We will keep a numeric tally of the number of occasions on which subjects did not participate and the reasons for non-participation (without personal identifiers).

4.6 Participant ID (PID) Assignment
Participants who give informed consent will have a unique participant ID number assigned, which will apply throughout the study.

4.7 Accrual
We plan to recruit men who request a circumcision to participate in the Unicirc data collection project.

5.0 PROJECT CONDUCT / PROCEDURES

5.1 Circumcision procedure
5.1.1 Evaluation prior to circumcision
Volunteers will be evaluated by a project physician prior to circumcision. A brief medical history will be obtained and history of possible bleeding disorder and medications likely to cause bleeding (e.g. aspirin) will be specifically elicited. A general exam will be conducted concentrating on examination of the genitalia. Men who have any penile abnormality or infection that could complicate the circumcision will be excluded, but may be offered circumcision outside of the study protocol. All of the relevant information will be recorded on eligibility checklists and reported on Case Report Forms (CRFs).

5.1.2 Risks of circumcision
Very rarely, patients may experience skin irritation from use of antiseptics prior to surgery. Adverse reactions to local anesthetics are not uncommon, but true allergy is very rare. Allergic reactions to the local

anesthetic are usually due to sensitivity to their metabolite, para-aminobenzoic acid (PABA). Non-allergic reactions may resemble allergy in their manifestations. There are also rare cases of allergy to paraben derivatives, which are often added as preservatives to local anesthetic solutions.

The risks of the procedure itself are found in Table 1.

In conclusion, this is a procedure, which has a high rate of minor complications (1% to 15%) and a very low rate of complications, which result in permanent damage. In over 1,000 circumcisions, the PI has never had a serious complication.

5.1.3 Circumcision techniques
Unicirc disposable instrument: the Unicirc is a disposable instrument that requires one hemostat, and a disposable scalpel to excise the prepuce. Only disposable surgical instruments will be used. The tissue adhesive comes in a single-use tube.

The circumcision procedure will be performed using standard sterile technique. Circumcised men will be observed for 30 minutes after the procedure, to make sure that there is minimal bleeding. Subjects will be given written post-operative instructions, including instructions to avoid sexual activity for 4 weeks following the procedure, and the telephone contact information of the doctor, so they can call with any concerns.

5.1.4 Post-procedure follow-up
Subjects will return for follow-up at 7 days and 4 weeks. Optionally, some sites may also choose to do follow-ups at 2 days and 14 days. In those men who have not completely healed by 4 weeks, we will conduct a 6 week follow-up visit to document complete healing. At each follow-up visit, a project doctor will exam the circumcision site and at some visits volunteers will be asked about pain, healing, satisfaction, and cosmetic result. Any complications will be noted on the CRF.

5.1.5 Complications. Participants will be able to contact the doctor who performed the circumcision at any time with questions or concerns via the on-call doctor's mobile phone. We will use a standardized surgical form to document the procedure and record any complications. We will grade complications (adverse events) as mild, moderate or severe, using the "PEPFAR Next Generation Indicators Reference Guide" (2009) (see Table 2). A standardized complication evaluation form will be completed at each of the 4 follow-up visits. Key variables will be anesthetic complications, bleeding, swelling, infection, and problems with urination, subsequent procedures conducted to correct complications, occupational exposure, and patient satisfaction. All complications will be noted on the CRF. Wound disruption is not considered a complication by PEPFAR, but will be recorded on the CRF. During unscheduled ill visits, all signs/symptoms, diagnoses, and medication(s) will be noted in the patient record and reported on CRFs. Should the ill visit constitute an adverse event (see Tables 1 and 2 for definitions), the Adverse Event form will be completed. Any ongoing concerns will be treated by the doctor.
Injury compensation will be provided in accordance with the Association of British Pharmaceutical Industry (ABPI) Guidelines.

Table 1. **Risk of male circumcision complications**

Author, year	Technique	N	Proceduralist	Risk of moderate-

Voluntary Adult Male Circumcision

				**severe complications***
Cummings, 2012	Forceps-guided	8487 adults	80% nurses, 20% doctors	0.76%
Tiwari, 2012	Open, closure with tissue adhesive or suture	88 adults and children	Urologist	Wound inflam 15.9%<br>Bleeding 4.5%<br>Infection 4.5%<br>Wound separation 3.4%
O'Sullivan,	Gomco, adhesive vs suture	100 adults and children	Surgeon	2.8% Gomco and adhesive<br>7.8% Gomco and suture
D'Arcy, 2011	Open, tissue adhesive	100 adults and children	Surgeon	7%
Auvert, 2005	Open	1546 adults	General practitioner	3.6%
Bailey, RC, 2007	Open	1391 adults	Doctors	1.5%
Gray, 2007	Open	2474	Doctors	4%

* Infections, pain, wound separation, hematomas or bleeding

Table 2. Classification and definitions of intra-operative and immediate post-operative adverse events following circumcision. (Source document: PEPFAR Next Generation Indicators Reference Guide, Vers 1.1, 2009 at www.pepfar.gov/documents/organization/81097.pdf)

For the specific moderate/severe AEs listed in the disaggregation above, the following guidance for distinguishing between moderate and severe is offered. Routine reporting of moderate and severe AEs is all that is recommended. AEs of seriousness less than moderate should not be reported.

ANESTHESIA REACTION:
-*Moderate*: Reaction to anesthetic requiring medical treatment on site, but not transfer to another facility (Palpitations, vaso-vagal reactions, or emesis would not qualify as moderate AE(s) unless such reaction(s) were so serious as to require medical treatment).

-*Severe*: Anaphylaxis or other reaction requiring hospitalization or referral/transfer to another facility

BLEEDING:
-*Moderate*: Intra-operative bleeding that requires a pressure dressing to control; or post-operative bleeding that requires a special return to the clinic for medical attention (Intra-operative bleeding that is easily controlled or post-operative spotting of the bandage with blood would not qualify as a moderate AE).
-*Severe*: Intra-operative bleeding requiring blood transfusion, transfer to another facility, or hospitalization; or post-operative bleeding that requires surgical reexploration, hospitalization, or transfer to another facility.

INFECTION:
-*Moderate*: Purulent discharge from the wound (Erythema around the incision line, by itself, would not be serious enough to qualify as a moderate AE)
-*Severe*: Cellulitis or wound necrosis

PAIN (INTRA- AND POST-OPERATIVE):
-*Moderate*: Pain serious enough to result in disability (as evidenced by loss of work or cancellation of normal activities) that lasting for at least 4 days after surgery but not more than 7 days

-*Severe*: Pain serious enough to result in disability (as evidenced by loss of work or cancellation of normal activities) lasting for at least 8 days after surgery. Pain that is so extraordinary as to result in early termination of surgery or administration of general anesthesia (where possible) would also be considered a severe pain AE.

WOUND DISRUPTION:
-*Moderate*: Surgical re-exploration is required, but hospitalization or referral to another facility is not necessary (Re-suturing, by itself, would not be considered serious enough to qualify as a moderate wound disruption AE)
-*Severe*: Referral/transfer to another facility or hospitalization is required.

SEXUAL DYSFUNCTION/UNDESIRABLE SENSORY CHANGES:
-*Moderate*: Post-operative changes that impair or preclude sexual function for between 3 and 6 months after the date of surgery (sexual dysfunction for a shorter period would not qualify as a moderate AE)
-*Severe*: Post-operative changes that impair or preclude sexual function for greater than 6 months after the date of surgery

SCARRING/DISFIGUREMENT/POOR COSMETIC RESULT; EXCESS SKIN REMOVAL; INJURY TO GLANS:
-Scarring/disfigurement/poor cosmetic result
Moderate: Scarring/disfigurement is discernible but re-operation not required (absence of discernible scarring/disfigurement, despite a client's complaint about the surgical outcome, would not be considered a moderate AE).
-Excess skin removal
Moderate: Tightening of the skin is discernible but reoperation not required (absence of discernible tightening of skin, despite a client's complaint about the surgical outcome, would not be considered a moderate AE).
-Injury to glans/shaft
Moderate: Abrasion of the glans or shaft requiring pressure dressing or additional surgical intervention to stop bleeding
-Scarring/disfigurement/poor cosmetic result
Severe: Requires re-operation or referral/transfer to another facility
-Excess skin removal Severe: Requires re-operation or referral/transfer to another facility
-Injury to glans/shaft Severe: Severing of the glans or shaft

OCCUPATIONAL EXPOSURE:
-*Moderate*: All occupational exposures are moderate (none are mild or severe)

OTHER: EXCESS SWELLING OF PENIS/SCROTUM (INCLUDING HEMATOMA); DIFFICULTY URINATING; OTHER:
-Excess swelling of penis/scrotum (including hematoma)
Moderate: Symptoms /signs so extraordinary as to cause disability (as evidenced by loss of work or cancellation of normal activities) lasting for at least 4 days after surgery but not more than 7 days.
-Difficulty urinating
Moderate: Partial obstruction requiring a special return to the clinic but no additional treatment (transient difficulty urinating that resolves on its own would not be considered a moderate AE).
-Other Moderate: Other adverse events related to the surgery that result in disability (as evidenced by loss of work or cancellation of normal activities) lasting for at least 4 days after surgery but not more than 7 days.

-Excess swelling of penis/scrotum (including hematoma)
Severe: Surgical re-exploration required or symptoms /signs so extraordinary as to cause disability (as evidenced by loss of work or cancellation of normal activities) lasting for at least 8 days after surgery
-Difficulty urinating
Severe: Complete obstruction and/or requires referral for treatment or surgery to correct.
-Other
Severe: Other AE(s) related to the surgery that result in disability (as evidenced by loss of work or cancellation of normal activities) lasting for at least 8 days after surgery, or result in hospitalization or referral/transfer to another facility.

5.2 Participants Lost to Follow-up
In case of absence at the scheduled follow-up visits, a project staff member will attempt to contact the subject on at least three occasions. Efforts will be made to keep all participants in project follow-up. A participant will not be classified as “lost to follow-up” until the end of the study.
Subjects who complete partial follow-up but then withdraw will be included in the data analysis to the point in which they were censored, and will be considered research subjects, with all the data protection elements of other subjects. For subjects who never complete the circumcision, the subject's name and all identifying information will be shredded and any data will be removed from the research database.

5.3 Off Project
Following are the reasons for discontinuation from project participation:
- Lost to follow-up
- Completion of project follow-up as defined by the protocol
- Premature closure of the project
- Refusal to continue any contact for follow-up by project participant
- Death of participant

When a participant goes Off Project, all data collection ends.

5.4 Data Reporting Requirements
For men who have undergone circumcision, the following reporting requirements will be used:
There will be a standardized surgical form, which will document the procedure and record any complications. With respect to grading of complications (adverse events) as mild, moderate or severe, we will use the PEPFAR Next Generation Indicators Reference Guide. A standardized evaluation form will be completed at each of the 2 follow-up visits. Questions will include the following (regardless of grade).
- Was there bleeding from the surgical site that required intervention?
  - Local pressure
  - Suture
  - Blood transfusion
  - Hospitalization
- Was there any sign/symptom of infection potentially related to the circumcision procedure? (Local or systemic)
  - Were antibiotics prescribed?
  - Was hospitalisation necessary?
- Were there any problems with urination?
  - If so, what; and was medical attention required?
- Did any other potentially procedure-related events occur?

- Was another surgical procedure required to correct the original procedure?
- Is the subject satisfied with the outcome of the procedure (with open-ended comments)?

General signs/symptoms/diagnoses that are definitely not related to the circumcision procedure will also be recorded, but will not be reported on CRFs, regardless of grade (unless Adverse Event reporting criteria are met—see below).

5.5 Serious Adverse Event reporting
The Standard Level of Serious Adverse Event reporting will be used according to the PEPFAR Next Generation Indicators Reference Guide. Serious complications will be reported to the project leadership within two (2) working days of becoming aware of the event:

- All disabilities/incapacities
- All hospitalizations that are "suspected adverse surgical reactions" (cannot rule out relationship to circumcision procedure)
- All other events defined as serious by the PEPFAR Next Generation Indicators Reference Guide that are "suspected adverse reactions" (cannot rule out relationship to circumcision procedure)

5.6 Laboratory test results
No laboratory tests will be routinely performed. Any laboratory test result that is obtained due to concern for medical or other illness (e.g. hemoglobin, etc.) will be reported, regardless of grade.

5.7 Participant remuneration

Neither the participant, nor his medical scheme will pay any study-based expenses.

6.0 STATISTICAL CONSIDERATIONS

6.1 General Design Issues
There are strong data to support the use of male circumcision in southern Africa to prevent acquisition of HIV infection, but there are very few data comparing various techniques.

6.2 Primary Outcome Measures

- Time required for procedure

6.3 Secondary Outcome Measures

- Complications (operative and post-operative); time to healing; direct costs; pain experienced during and after the procedure; patient satisfaction; cosmetic result.

We expect the circumcision-related minor-moderate complication rate to be less than 10%. We expect the circumcision-related serious Adverse Event rate to be less than 1%.

If it is unclear whether an outcome represents a procedure-related event for purposes of reporting/analysis, its status as such will be decided by an independent surgeon.

Outcome measures will be measured at every follow-up visit, except the physician questionnaire, which will be conducted once at the end of the study.

6.4 Sample Size

UNICIRC Protocol Phase 4 research data collection: **Application for a registry**.

Voluntary Adult Male Circumcision

According to the "Framework for Evaluation of Adult Circumcision Devices (WHO, 2011): "While the incidence of adverse events and device-related incidents are important in the assessment of the devices, other outcomes should be considered primary endpoints and drive the sample size requirements. Studies involving about 100 men (range 50 to 300) are suggested as a compromise between assessing safety, documenting the presumed advantages of the new method, and ensuring rapid progress through the different stages of clinical assessment…."

6.5 Stopping rules:
The study will be stopped if serious complications are observed in 5% men circumcised.

6.6 Data Collection and Management
We will collect data on demographics; socioeconomic status; circumcision knowledge/attitudes; time and degree of difficulty learning and performing the surgery; cost of materials; complications; patient perceived pain during and after the procedure, degree of patient satisfaction, and cosmetic result. We will use standardized Case Report Forms (CRFs) to collect data on potential confounders at baseline, and primary and secondary outcomes during surgery and at follow-up visits. Qualified data management personnel will enter and manage data using standard database procedures. CRFs and data will be kept in locked areas and password protected.

6.6 Analysis
We will conduct: 1) analysis of baseline data to examine potential confounders; 2) calculation of descriptive statistics of outcomes; and 3) statistical tests of differences in scale (e.g. Likert), time, cost, and proportions (relative risk) for outcome variables.

6.7 Publication
The doctors performing circumcisions will play no role in data management, data analysis, or reporting of results. They will have no decision-making powers with regard to dissemination of the results. The study will be published, or the results disseminated via clinicaltrials.gov, regardless of the study results.

7.0 HUMAN SUBJECTS CONSIDERATIONS

7.1 Protection of Human Subjects
This research study will comply with all principles contained in the Declaration of Helsinki and the South African Department of Health Clinical Trial and Ethics in Health Research Guidelines. We will adhere to the ethical principles and guidelines for the protection of human subjects as detailed in the Belmont Report.

With regard to respect for persons, we will ensure that the participants will receive thorough and accurate information about the potential risks and benefits of circumcision. Each subject will be given written informed consent prior to circumcision. The informed consent will include detailed descriptions of the risks and benefits. It will be in English, and in the 2 most common local languages (Xhosa and Afrikaans). The consent form will be read aloud to men with poor literacy skills. Subjects will be given an opportunity to read or to have the informed consent form read to them, and to discuss with their family or partner before making a decision as to whether or not to participate in the study.

With regard to beneficence, we believe that given the current state and future prospects of the HIV epidemic, the evidence of the protective effect of male circumcision on HIV infection, and the evidence that male circumcision has health benefits, the benefits of male circumcision outweigh the risks of this procedure.

7.2 Confidentiality
Questions will center on the results of the circumcision with some questions on resumption of sexual practice after circumcision, but not on past sexual history. Moreover, circumcision is a confidential procedure and confidentiality will be assured at all times by conducting interviews and questionnaires in a private area, and keeping all study material locked in a filing cabinet at all times. Any telephone calls made to subjects to ensure follow-up will not mention the specific reason for the call or reveal any confidential information.

8.0 BIOHAZARD CONTAINMENT / INFECTION CONTROL
As the transmission of HIV and other blood-borne pathogens can occur through contact with contaminated needles, blood, and blood products, appropriate blood and secretion precautions will be employed by all personnel. Only disposable surgical instruments will be used.

OUTLINE OF PROCEDURES
Recruitment of subjects:
- Publicity campaign

Determination of eligibility:
- An in-person interview

For subjects potentially qualifying:
- Informed consent
- In-person interview
- Eligibility requirements reviewed and confirmed
- Genital exam to rule out abnormalities of the prepuce

For subjects recruited into the study:
- Demographics questionnaire
- Date scheduled for circumcision

For subjects arriving for circumcision
- All eligibility requirements reviewed and met
- Informed consent for Circumcision with Unicirc Instrument and tissue adhesive
- Surgeon completes surgical CRF
- Post-op instructions given
- First follow-up visit scheduled

Optional follow-up visit, 2 days:
- Physical examination of the circumcision
- CRF completed by examining physician
- Interview completed by subject
- Second follow-up visit scheduled

Follow-up visit, 7 days
Physical examination of the circumcision
- CRF completed by examining physician
- Interview completed by subject
- Third follow-up visit scheduled

Optional follow-up visit, 14 days
Physical examination of the circumcision
- CRF completed by examining physician

UNICIRC Protocol Phase 4 research data collection: Application for a registry.

Voluntary Adult Male Circumcision

- Interview completed by subject
- 4th follow-up visit scheduled

Follow-up visit, 28 days
Physical examination of the circumcision

- CRF completed by examining physician
- Interview completed by subject
- Subject discharged from study at 4 weeks, assuming healing and no complications
- If circumcision is not fully healed, we will conduct a follow-up visit 2 weeks later

UNICIRC Protocol Phase 4 research data collection:
Application for a registry.

Voluntary Adult Male Circumcision

<u>Follow-up visits, 28 days (if not fully healed at 4 weeks)</u>

- Physical examination of the circumcision
- CRF completed by examining physician
- Subject discharged from study

References:

1. Zafar F, Thompson JN, Pati J, Kiely EA, Abel PD. Sutureless circumcision. The British journal of surgery. 1993 Jul;80(7):859. PubMed PMID: 8369915.
2. Millard PS. Rapid, minimally invasive adult voluntary male circumcision: a randomized trial. S Afr Med J. 2013;In press.
3. Considerations for implementing models for optimizing the volume and efficiency of male circumcision services. Geneva: World Health Organization; 2010.
4. Gray R, Kigozi G, Serwadda D, Makumbi F, Watya S, Nalugoda F, et al., editors. Randomized Trial of Male Circumcision for HIV Prevention in Rakai, Uganda, abstract 155aLB. 14th Conference on Retroviruses and Opportunistic Infections; 2007; Los Angeles, California, USA.
5. Manual for Male Circumcision under Local Anaesthesia Geneva: World Health Organization; 2009.
6. Bailey RC, Egesah O, Rosenberg S. Male circumcision for HIV prevention: a prospective study of complications in clinical and traditional settings in Bungoma, Kenya. Bull World Health Organ. 2008 Sep;86(9):669-77. PubMed PMID: 18797642. Pubmed Central PMCID: 2649497. Epub 2008/09/18. eng.
7. Kim HH, Goldstein M. High complication rates challenge the implementation of male circumcision for HIV prevention in Africa. Nat Clin Pract Urol. 2009 Feb;6(2):64-5. PubMed PMID: 19107114. Epub 2008/12/25. eng.
8. US Food and Drug Administration. Avoiding Patient Injuries from Circumcision Clamps 2002. Available from: http://www.accessdata.fda.gov/scripts/cdrh/cfdocs/psn/printer.cfm?id=92.
9. Lane V, Vajda P, Subramaniam R. Paediatric sutureless circumcision: a systematic literature review. Pediatr Surg Int. 2010 Feb;26(2):141-4. PubMed PMID: 19707772. Epub 2009/08/27. eng.
10. Elmore JM, Smith EA, Kirsch AJ. Sutureless circumcision using 2-octyl cyanoacrylate (Dermabond): appraisal after 18-month experience. Urology. 2007 Oct;70(4):803-6. PubMed PMID: 17991565. Epub 2007/11/10. eng.
11. Kaye JD, Kalisvaart JF, Cuda SP, Elmore JM, Cerwinka WH, Kirsch AJ. Sutureless and scalpel-free circumcision--more rapid, less expensive and better? J Urol. 2010 Oct;184(4 Suppl):1758-62. PubMed PMID: 20728122. Epub 2010/08/24. eng.
